# Functional characterization of urine-derived stem cells from acute-on-chronic liver failure patients in an immune-mediated acute liver injury model

**DOI:** 10.3389/fbioe.2026.1759241

**Published:** 2026-04-17

**Authors:** Jiateng Zhang, Pengfei Yu, Jiaqi Li, Xiao Lin, Rui Zhao, Huaibin Zou, Yu Chen, Yuanyuan Zhang, Zhongping Duan

**Affiliations:** 1 Fourth Department of Liver Disease (Difficult and Complicated Liver Diseases and Artificial Liver Center), Beijing Youan Hospital, Capital Medical University, Beijing, China; 2 Beijing Key Laboratory of Liver Regeneration and Artificial Liver Transformation Research, Beijing, China; 3 Second Department of Liver Disease, Beijing Youan Hospital, Capital Medical University, Beijing, China; 4 Wake Forest Institute for Regeneration Medicine, Wake Forest School of Medicine, Winston-Salem, NC, United States

**Keywords:** acute-on-chronic liver failure, cell transplantation, inflammation, stem cell therapy, urine-derived stem cells

## Abstract

**Introduction:**

Acute-on-chronic liver failure (ACLF) is a highly lethal clinical syndrome with limited effective therapeutic options. Urine-derived stem cells (USCs) represent a non-invasive and readily accessible cell source, but whether USCs obtained from patients with severe liver dysfunction retain therapeutic and immunomodulatory potential remains unclear.

**Methods:**

To address this question, USCs derived from ACLF patients (LF-USCs) were evaluated in a Concanavalin A (Con A)-induced immune-mediated acute liver injury mouse model. Hydrogel-encapsulated LF-USCs were transplanted, and therapeutic efficacy was assessed by survival analysis, serum biochemical parameters, histological examination, and inflammatory cytokine profiling.

**Results:**

Transplantation of hydrogel-encapsulated LF-USCs significantly improved mouse survival, reduced serum transaminase levels, and alleviated hepatocellular necrosis (p < 0.05). At the mechanistic level, LF-USC treatment was associated with decreased systemic inflammatory cytokine levels, attenuation of intrahepatic inflammatory injury, and dynamic modulation of macrophage-associated inflammatory signatures.

**Discussion:**

These findings demonstrate that functionally competent USCs can be successfully obtained from ACLF patients and highlight their potential as a readily accessible autologous cell source for immune modulation and liver tissue repair in immune-mediated acute liver injury.

## 1 Introduction

Liver failure is a severe clinical syndrome caused by multiple etiologies, characterized by acute onset, rapid progression, and high mortality, and thus poses a major threat to human health (Stravitz et al., 2023). A central pathological feature of liver failure is the excessive inflammatory response, in which uncontrolled cytokine release and aberrant immune cell activation drive extensive hepatocyte necrosis and rapid hepatic decompensation (Qiang et al., 2022; Lu et al., 2025).

Within the spectrum of liver failure, acute liver failure (ALF) has been widely used to investigate immune-mediated mechanisms because of its abrupt onset and prominent inflammatory features (Fernández et al., 2024). Among experimental models, the Concanavalin A (Con A)-induced murine acute liver injury model is a well-established immune-mediated hepatitis model that effectively recapitulates T-cell activation and cytokine storm-driven hepatic injury (Ibrahim et al., 2021; Bi et al., 2019; Liu X. et al., 2021). In contrast, acute-on-chronic liver failure (ACLF) represents a distinct clinical entity characterized by acute deterioration superimposed on chronic liver disease and is associated with extremely poor prognosis and limited therapeutic options (Bajaj et al., 2022; ABBAS et al., 2022). The key to blocking the transition to severe ACLF lies in neutralizing the acute liver insult and its powerful inflammatory aftermath (European Association for the Study of the Liver, 2023; Artru and Mcphail, 2024).

In recent years, stem cells have emerged as a promising therapeutic approach owing to their potent immunomodulatory and regenerative properties (Huang et al., 2022; Zhidu et al., 2024). Among them, urine-derived stem cells (USCs) have garnered particular attention because they can be obtained non-invasively, are widely accessible, and display favorable biological characteristics (Hu et al., 2021; Atia et al., 2025). Nevertheless, studies specifically addressing urine-derived stem cells from ACLF patients (LF-USCs) remain scarce, and their mechanisms of action and clinical feasibility in the treatment of liver failure are largely unknown. To address this gap, the present study employed a Con A-induced murine ALF model to investigate the anti-inflammatory and hepatoprotective effects of LF-USCs. These findings are expected to provide experimental evidence supporting LF-USCs as a potential autologous cell therapy and to lay the groundwork for clinical translation in liver failure.

## 2 Materials and methods

### 2.1 Isolation and expansion of USCs

LF-USCs were isolated from urine samples collected from three male patients diagnosed with acute-on-chronic liver failure (ACLF) at Beijing Youan Hospital, Capital Medical University. Primary baseline clinical characteristics of the ACLF patients are summarized in Supplementary Table S1. Urine samples were collected after clinical diagnosis of ACLF and during routine inpatient management, before experimental cell isolation. USCs from age-matched healthy donors (H-USCs) (n = 3) were obtained as controls. Healthy donors were volunteers without a history of liver disease, chronic systemic illness, or recent acute infection. Urine samples from healthy donors were collected by spontaneous midstream urination and processed using the same protocol as that applied to ACLF patient samples. For each patient, at least three urine samples (ranging from 100 to 300 mL) were collected during the initial stage. These samples were processed independently for USC isolation rather than pooled. The resulting USC cultures were cryopreserved as individual batches and subsequently thawed and expanded as needed for downstream experiments. Sterile urine samples were first centrifuged at 1000–1500 rpm for 3–5 min, after which the supernatant was discarded, leaving 1–2 mL of pellet. The pellet was resuspended in approximately 5 mL phosphate-buffered saline (PBS), centrifuged again under the same conditions, washed, and finally resuspended in USC-specific culture medium. The resulting cell suspension was seeded into T25 culture flasks and kept undisturbed for the first 48 h, after which the medium was replaced every 2 days. After approximately 2 weeks, when large or multiple colonies appeared, cells were subcultured. All cells were maintained under standard culture conditions (37 °C, 5% CO_2_), and cells within six passages (P6) were used for subsequent experiments, except for proliferation assays. Detailed cell culture procedures are described in Supplementary Method S1.

This research protocol was approved by the Ethics Committee of Beijing Youan Hospital, Capital Medical University (LL-2020–178-K). Written informed consent was obtained from all participating patients for the use of their medical records.

### 2.2 Cell activity and proliferation

Cell proliferation of LF-USCs was evaluated by both CCK-8 growth curve analysis and PD kinetics (Fan et al., 2024). For the CCK-8 assay, gradient-diluted cell suspensions were seeded into 96-well plates with six replicate wells per group. After a 2–4 h incubation for cell adherence, CCK-8 reagent was added, and cells were further cultured. Absorbance (O.D.) values were measured to generate a standard curve. Based on this curve, cells at the lowest concentration were seeded in six replicate wells and pre-incubated at 37 °C with 5% CO_2_. CCK-8 reagent was added every 24 h for six consecutive days, and O.D. values were recorded to calculate cell numbers.

For PD curve analysis, passage 1 LF-USCs were seeded at 1 × 10^6^ cells per 60-mm dish (N_1_) and sub-cultured every 3 days (CT) at the same density up to passage 10. Cell counts at each passage (N_n_) were obtained using a cell counter. PD was calculated as log_2_(N_n_/N_1_), and DT was derived as DT = CT/PD. All experiments were independently performed in triplicate.

### 2.3 Flow cytometry phenotyping of USCs

Flow cytometry was performed to characterize LF-USC surface markers (CD73^+^, CD90^+^, CD105^+^; CD34^−^, CD45^−^, CD11b^−^) using the OriCell® Human MSC Surface Marker Analysis Kit (Cat. No. HUXMX-09011) and an anti-CD90 antibody (Bioss, bsm-30105M), following the manufacturer’s protocols. Briefly, LF-USCs at 70%–80% confluence were detached, harvested, and resuspended in flow cytometry buffer (1× PBS with 0.1% BSA) at 3 × 10^6^ cells/mL. Cells were incubated with the respective primary antibody at 4 °C in the dark for 30 min, washed, and subsequently incubated with fluorescent secondary antibodies under the same conditions. After two final washes, cells were resuspended in flow buffer and immediately analyzed by flow cytometry. Detailed information on the antibodies used is listed in Supplementary Table S2.

### 2.4 USC trilineage differentiation

LF-USCs at passages P3-P5 were used for trilineage differentiation assays. Trilineage differentiation capacity was validated in cells derived from independent donors, and representative results are presented. For chondrogenic induction, 3–4 × 10^5^ cells were transferred to a 15 mL centrifuge tube for induction. For osteogenic and adipogenic induction, cells were seeded in six-well plates at a density of 2 × 10^4^ cells/cm^2^. Differentiation was performed using OriCell® kits (chondrogenic: HUXXC-90041; osteogenic: HUXXC-90021; adipogenic: HUXXC-90031, China) according to the manufacturer’s instructions.

### 2.5 Animal model and cell transplantation

Male (Liu et al., 2022) C57BL/6J mice (10 weeks old; Sipeifu, China) were acclimated under sterile conditions for 2 weeks before experiments. At 12 weeks of age, mice were randomly assigned to four groups: normal control (WT), model control (No-cell-18h post Con A), treatment group 1 (LF-USC-18h post Con A), and treatment group 2 (LF-USC-72h post Con A). Acute hepatitis was induced in the model and treatment groups by a single intravenous injection of concanavalin A (Con A, 35 mg/kg) (Bai et al., 2023; Huang et al., 2014). Four hours later, treatment groups received intraperitoneal administration of ∼2 × 10^6^ LF-USCs encapsulated in high-concentration RGD-modified hydrogel (XPBioMed, TWG003, China; 200 μL), while model controls received an equal volume of PBS in hydrogel. For survival analysis, the sample sizes were PBS model control, n = 14; LF-USC treatment groups combined, n = 22. The larger allocation to the LF-USC groups was intentional to compensate for the high and variable early mortality associated with the Con A model and to ensure sufficient statistical power for survival comparisons. Liver and serum samples were collected from surviving mice at 18 h (model control and treatment group 1) and 72 h (treatment group 2). Each experimental group consisted of six mice (n = 6). All procedures were approved by the Animal Ethics Committee of Capital Medical University (AEEI-2025–408).

### 2.6 Cell viability assay

Hydrogel encapsulation technology can mitigate immune rejection and enhance transplanted cell survival (Zhang et al., 2022; Lan et al., 2020). In this study, cells were encapsulated in high-concentration RGD-modified hydrogel (XPBioMed, TWG003, China) and administered via intraperitoneal injection to evaluate their therapeutic efficacy. Cell viability was evaluated with the Cyto3D™ Live/Dead Cell Viability Assay Kit (BM01, TWG003, China) according to the manufacturer’s protocol, and fluorescence images were acquired using a Nikon microscope (Nikon, Japan).

### 2.7 Serum biochemical analysis and inflammatory cytokine analysis

Serum levels of ALT, AST, and TBiL were measured using a Chemray 240 automated biochemical analyzer (Shenzhen Rayto Life and Analytical Sciences, China) following the manufacturer’s protocol.

Inflammatory cytokine levels in mouse serum and liver tissues were quantified using ELISA kits (Thermo Fisher Scientific, United States) following the manufacturer’s protocol. For specific information on ELISA kits, see Supplementary Table S3.

### 2.8 Histology and immunofluorescence staining

Liver tissues were fixed in 4% paraformaldehyde, paraffin-embedded, and sectioned for histological analysis. Sections were stained with hematoxylin and eosin (HE; Servicebio, G1005, China), periodic acid-Schiff (PAS; Servicebio, G1008, China), and Masson’s trichrome (Servicebio, G1006, China), and images were acquired using a Nikon upright optical microscope (Japan). Apoptosis was assessed by TUNEL staining (Servicebio, G1504, China) following the manufacturer’s protocol.

For immunofluorescence and immunohistochemical, sections were deparaffinized, hydrated, and subjected to antigen retrieval using EDTA buffer (pH 9.0) or citrate buffer (pH 6.0) at 95 °C for 20 min. Endogenous peroxidase was blocked with 3% hydrogen peroxide for 25 min, and nonspecific binding was minimized with 3% goat serum for 30 min. Sections were incubated with primary antibodies overnight at 4 °C, followed by HRP-conjugated secondary antibodies at room temperature for 50 min. Tyramide Signal Amplification (TSA) was performed in the dark for 10 min. After microwave-mediated stripping removed antibodies while retaining the fluorophore-labeled tyramide, a second primary antibody was applied, and the procedure was repeated. Nuclei were counter-stained with DAPI, autofluorescence was quenched, and sections were mounted. Antibodies included F4/80 (Servicebio, GB113373, 1:5000), TNF-α (Servicebio, GB115702, 1:5000), CD206 (Servicebio, GB113497, 1:4000), CD86 (Servicebio, GB150054, 1:5000)and HRP-conjugated goat anti-rabbit IgG (Servicebio, GB23303, 1:500); CD3 (Servicebio, GB150004, 1:1000), CD68 (Servicebio, GB153109, 1:3000) and S-vision Immunohistochemistry Polyclonal Antibody (Goat Anti-Rabbit) (Servicebio, G1302, Ready-to-use). Detailed information on the antibodies used is listed in Supplementary Table S4.

For histological and immunohistochemical analyses, three mice were randomly selected from each experimental group (6 mice per group), and liver tissue sections were prepared for staining. All liver tissue sections were cut at a thickness of 5 μm to ensure optimal staining quality and consistent imaging.

### 2.9 Reverse Transcription Quantitative Polymerase Chain Reaction (RT-qPCR)

Total RNA was extracted from liver tissues using the FastPure Cell/Tissue Total RNA Isolation Kit V2 (Nanjing Vazyme Biotech, China) according to the manufacturer’s protocol. RNA was reverse-transcribed into cDNA using the PrimeScript RT Master Mix (TaKaRa, Japan). RT-qPCR was performed with TB Green Premix Ex Taq II (Tli RNaseH Plus; TaKaRa, Japan), using GAPDH as an internal reference. Primer sequences for all genes are listed in Supplementary Table S5.

### 2.10 Western blot analysis

Total protein was extracted from liver tissues using RIPA lysis buffer (Thermo Fisher Scientific, United States) and analyzed by Western blot according to standard protocols. Equal amounts of protein (50 µg per lane) were loaded for SDS-PAGE analysis. Detailed information on the antibodies used is provided in Supplementary Table S6.

### 2.11 Bulk RNA-seq

Mouse liver tissues from the No-cell-18 h post Con A group (A group, n = 5) and LF-USC-72 h post Con A group (B group, n = 4) were collected for bulk RNA sequencing. Total RNA was extracted using TRIzol reagent, and samples with RNA integrity number≥5.0 were subjected to library preparation and sequencing by LC-Bio Technologies (Hangzhou, China) on the Illumina NovaSeq 6000 platform (2 × 150 bp). Clean reads were obtained using FastQC, aligned to the mouse reference genome (GRCm39) with HISAT2, and quantified using StringTie. Differentially expressed genes between groups were identified by DESeq2, with significance thresholds set at |log2FC|≥1 and adjusted *p* < 0.05. Functional enrichment analyses (GO and KEGG) and visualization were performed in R.

### 2.12 Statistical analysis

All data are presented as mean ± standard error of the mean (SEM). Survival data were analyzed using Kaplan-Meier curves with log-rank tests. For comparisons involving two groups, unpaired two-tailed Student’s t-tests were applied. For multiple group comparisons, one-way analysis of variance (ANOVA) followed by appropriate *post hoc* tests was used. Sample sizes for each experimental group are indicated in the Methods and figure legends. Statistical analyses were performed using R software (version 4.4.3), and p < 0.05 was considered statistically significant.

## 3 Results

### 3.1 Isolation, morphology, and cell growth characteristics of LF-USCs

**FIGURE 1:**
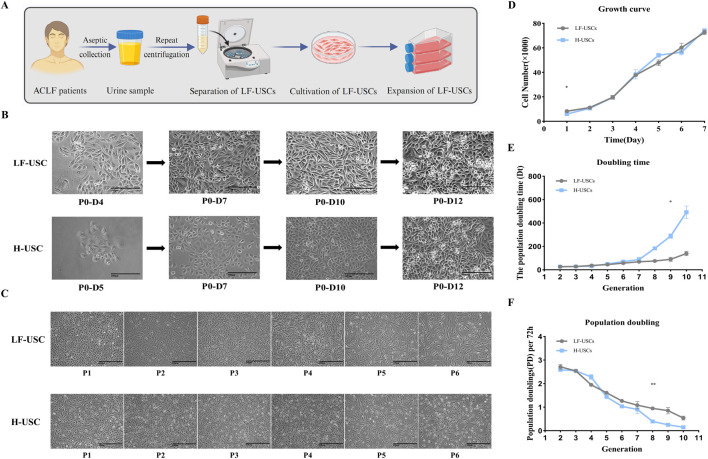
Isolation, morphology, and growth characteristics of LF-USCs. USCs for healthy donors (H-USCs) were used as a control. **(A)** Schematic workflow of LF-USCs isolation from the urine of ACLF patients. The separation process for H-USCs is the same as above. Created in BioRender. Yu, P. (2026). https://BioRender.com/u6oufbr. **(B)** Representative morphological images of primary LF-USCs/H-USCs at P0 (Day 4/5, Day 7, Day 10, Day 12; the magnification is ×200). Scale bar = 100 μm. **(C)** Morphology of LF-USCs/H-USCs during passages 1 to 6 (100×). Scale bar = 100 μm. **(D)** Proliferation curve of LF-USCs/H-USCs under 2D culture conditions measured by CCK-8 assay. Cell proliferation was measured using the CCK8 assay. Absorbance values were converted to cell numbers using a standard curve, and the Y-axis represents the corresponding cell number. **(E)** Doubling time (Dt) curve showing growth kinetics of LF-USCs/H-USCs (P2-P10). **(F)** Population doubling (PD) showing growth kinetics of LF-USCs/H-USCs (P2-P10). The data in panels **(D–F)** are presented as means ± standard error of the mean (SEM), with statistical significance indicated by asterisks. **p < 0.01. *p < 0.05.

Sterile urine samples from ACLF patients were centrifuged to collect the cellular sediment, which was subsequently resuspended in culture medium to establish LF-USCs (Figure 1A). H-USCs were obtained as a control. Primary USCs (P0) adhered to the culture surface within 2–3 days, and clonal colonies became visible between days 4 and 9. These colonies exhibited a uniform “rice grain”-like morphology and consistent size (Figure 1B). By days 10–14, larger clusters had developed, enabling subsequent passaging. With routine passaging, cells demonstrated accelerated proliferation, reaching 80%–90% confluence within 3–4 days (Figure 1D). However, at higher passage numbers, cells gradually transitioned from the initial “rice grain” morphology to a spindle-shaped phenotype, accompanied by increased cell size and a pronounced decline in proliferative capacity (Figures 1C,E,F). Additionally, using H-USCs as a control, the growth curves of both the LF-USCs group and the H-USCs group were substantially aligned (Figure 1D).

We analyzed the cell proliferation characteristics of LF-USCs and H-USCs across ten consecutive passages (P1-P10). No significant differences were observed in the Doubling Time (DT) or Cumulative Population Doubling (PD) between the two groups at each passage level, except for passages P8 and P9 (Figures 1E,F). These findings suggest that the LF-USCs maintain cellular morphology and proliferative capacity highly similar to those of H-USCs throughout the early and intermediate passages. The increased doubling time suggests the patient USCs are less robust and have a shorter proliferative lifespan in culture than those from healthy donors after passage 7, which is a critical consideration for clinical applications requiring cells at early passage.

### 3.2 Phenotypic and differentiation characterization of LF-USCs and H-USCs

**FIGURE 2:**
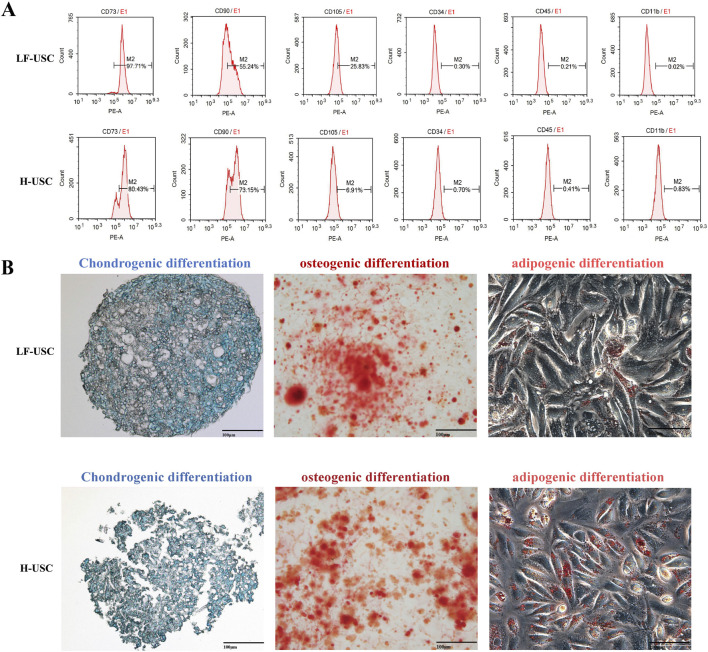
Phenotypic and differentiation characterization of LF-USCs and H-USCs. **(A)** Flow cytometry analysis showing positive expression of MSC markers (CD73, CD90, CD105) and negative expression of hematopoietic markers (CD34, CD45, CD11b). **(B)** Multilineage differentiation potential of USCs demonstrated by chondrogenesis (Alcian Blue staining), osteogenesis (Alizarin Red staining), and adipogenesis (Oil Red O staining). Scale bar = 100 μm. The magnification is ×200.

The equations should be inserted in an editable format from the equation editor. Flow cytometry analysis demonstrated that LF-USCs and H-USCs expressed typical mesenchymal stem cell markers (CD73, CD90, CD105) while lacking hematopoietic lineage markers (CD34, CD45, CD11b) (Sun et al., 2024; Wu et al., 2021) (Figure 2A). Moreover, LF-USCs and H-USCs exhibited multipotent differentiation potential, as evidenced by successful induction into chondrogenic, osteogenic, and adipogenic lineages, confirmed by Alcian Blue, Alizarin Red, and Oil Red O staining, respectively (Figure 2B). These data indicate that we have successfully isolated and cultured USCs, which exhibit characteristic features similar to mesenchymal stem cells and demonstrate certain multipotent differentiation capabilities (Boysen et al., 2024; Wang et al., 2024).

### 3.3 LF-USCs rescue of Con A-induced liver failure

**FIGURE 3:**
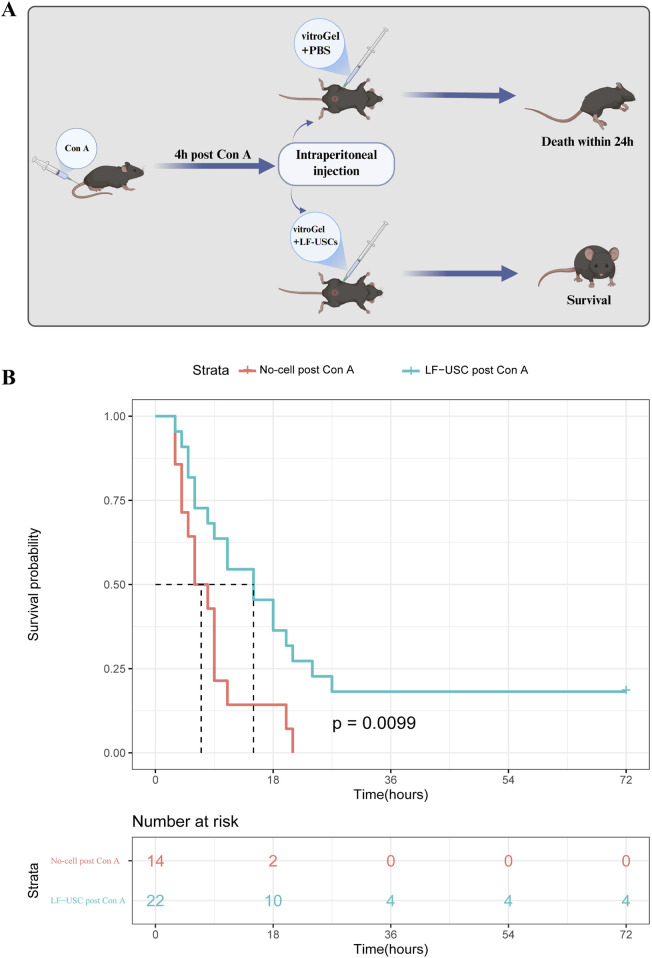
LF-USCs rescue of concanavalin A (Con A)-induced liver failure. **(A)** Schematic diagram of LF-USCs transplantation in the Con A model. Created in BioRender. Yu, P. (2026). https://BioRender.com/lwi9qek. **(B)** Kaplan-Meier survival curves for Con A-induced acute liver failure mice following injection of PBS (n = 14), LF-USCs (n = 22). Significance was determined by comparison with the PBS group using a log-rank test (p < 0.01).

A mouse model of acute liver injury was established by Con A-mediated activation of natural killer T cells (Liu et al., 2022) and subsequently employed to evaluate the therapeutic efficacy of LF-USCs (Figure 3A). Kaplan-Meier survival analysis showed that mice receiving Con A followed by intraperitoneal PBS injection succumbed within 24 h due to fulminant hepatic failure, whereas LF-USC-transplanted mice exhibited significantly improved survival, with benefits extending over 72 h (Figure 3B, p < 0.01). These results indicate that LF-USC transplantation markedly enhances survival outcomes in Con A-induced acute liver failure.

### 3.4 Cell viability of LF-USCs is encapsulated in a hydrogel

**FIGURE 4:**
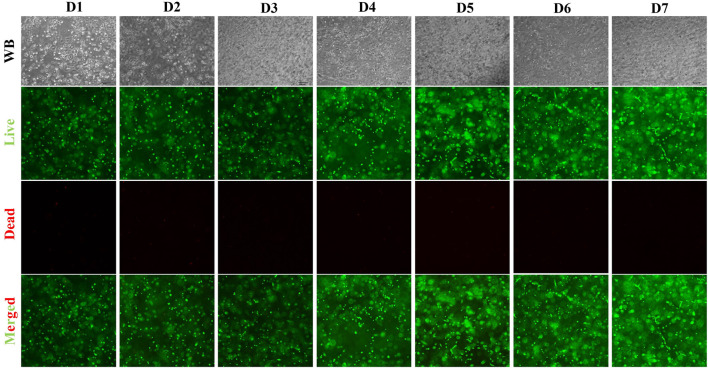
Cell viability of LF-USCs encapsulated in hydrogel. Live/dead staining images (100×) showing the viability of LF-USCs encapsulated in hydrogel for up to 7 days. Scale bar = 100 μm.

Live/dead staining confirmed that LF-USCs encapsulated in hydrogels maintained robust viability and proliferative activity for up to 7 days (Figure 4), further supporting the feasibility of this transplantation strategy (Liu and Hong, 2024).

### 3.5 LF-USCs transplantation attenuates hepatocyte injury and promotes hepatic recovery in Con A-induced ALF mice

**FIGURE 5:**
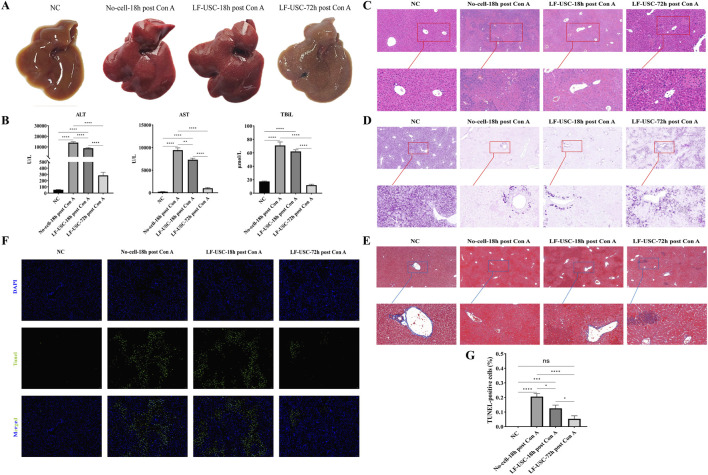
LF-USCs transplantation attenuates liver injury in Con A-induced ALF mice. **(A)** Typical morphological features of each group of livers are shown in figure. **(B)** The levels of the liver function indexes (ALT, AST, and TBiL) were measured. n = 6 mice per group. **(C)** Representative images of the liver subjected to H&E staining. The magnification is ×200 and 400×. **(D)** Representative images of the liver subjected to PAS staining (100× and 400×). **(E)** Representative images of the liver subjected to Masson staining (100× and 400×). Scale bar = 100 μm. **(F)** Representative images of the liver subjected to TUNEL staining (200×) and **(G)** quantitative analysis of apoptotic areas in liver tissue cells per group. **(C–F)** Three mice were randomly selected from each group (n = 6), and **(G)** three random fields per section were analyzed for TUNEL quantification. Scale bar = 50 μm. The data in panels B and G are presented as means ± standard error of the mean (SEM), with statistical significance indicated by asterisks. ****p < 0.0001. ***p < 0.001. **p < 0.01. *p < 0.05.

To directly assess hepatocyte injury and recovery, macroscopic, functional, and histological analyses were performed following LF-USC transplantation in Con A-induced ALF mice. Macroscopic examination of mouse livers revealed that the normal control group exhibited smooth surfaces, uniform coloration, and preserved morphology. In contrast, both the No-cell-18h post Con A group and the LF-USC-18h post Con A group showed markedly swollen livers with fragile texture and extensive hemorrhagic necrosis, indicative of severe hepatic injury. By comparison, the LF-USC-72h post Con A group displayed clear signs of recovery, with reduced congestion and necrosis relative to the untreated group, suggesting that LF-USC transplantation facilitated progressive hepatic repair (Figure 5A). Consistent with these morphological observations, serum alanine aminotransferase (ALT), aspartate aminotransferase (AST), and total bilirubin (TBiL) levels were significantly elevated in the No-cell-18h post Con A group but markedly decreased following LF-USC treatment, demonstrating substantial restoration of liver function (Figure 5B). Histological analyses further substantiated hepatocyte protection and recovery. Hematoxylin and eosin staining revealed extensive hepatocellular necrosis and architectural disruption in untreated mice, whereas LF-USC-treated livers, particularly at 72 h, displayed preserved lobular architecture and reduced necrotic regions (Figure 5C). Periodic acid Schiff (PAS) staining showed severe depletion of hepatic glycogen stores following Con A challenge, which was partially restored in LF-USC-treated mice, indicating recovery of hepatocyte metabolic capacity (Figure 5D). Masson’s staining showed marked hepatic structural disruption with prominent inflammatory cell infiltration in the No-cell-18 h post Con A group, without extensive collagen deposition. In contrast, LF-USC transplantation improved hepatic architecture, and the LF-USC-72 h post Con A group exhibited largely preserved lobular structure with only limited, focal collagen fibers (Figure 5E). In parallel, TUNEL staining demonstrated a pronounced reduction in apoptotic hepatocytes after LF-USC treatment, confirming effective attenuation of hepatocyte death (Figures 5F–G). Collectively, these findings demonstrate that LF-USC transplantation not only mitigates acute liver injury but also directly protects hepatocytes and promotes structural and functional hepatic recovery in Con A-induced ALF mice.

### 3.6 LF-USCs modulate inflammatory responses in Con A-induced ALF mice

**FIGURE 6:**
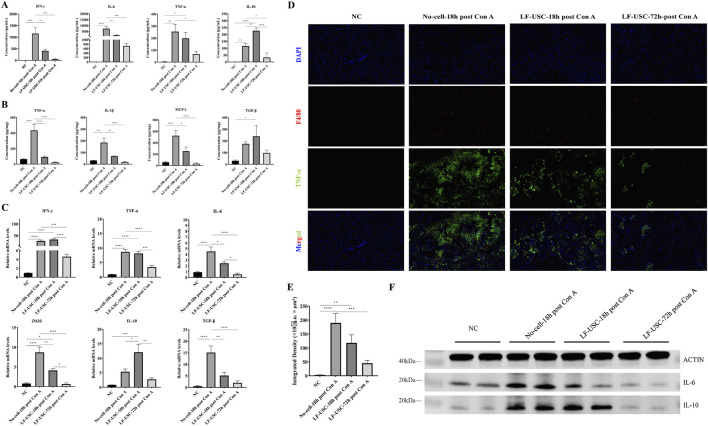
LF-USCs transplantation alleviates hepatic inflammation. ELISA assay for inflammatory factor levels in serum **(A)** and liver tissue homogenates **(B)** from each group (n = 6). **(C)** RT-qPCR analysis of inflammatory gene expression in liver tissues (n = 6). **(D)** Immunofluorescence staining of liver sections showing macrophage marker F4/80 and TNF-α. Scale bar = 50 μm. **(E)** Quantitative analysis of total TNF-α expression within positive regions of liver tissue in each group. Three mice were randomly selected from each group (n = 6), and three random fields per section were analyzed for quantification. **(F)** Western blot analysis of IL-6 and IL-10 protein expression in each group. The data in panels **(A–C,E)** are presented as means ± standard error of the mean (SEM), with statistical significance indicated by asterisks. ****p < 0.0001. ***p < 0.001. **p < 0.01. *p < 0.05.

Upon Con A treatment, the inflammatory response in mice was significantly enhanced, as demonstrated by elevated levels of proinflammatory cytokines, including interferon-γ (IFN-γ), interleukin-6 (IL-6), tumor necrosis factor-α (TNF-α), interleu-kin-1β (IL-1β), and monocyte chemoattractant protein-1 (MCP-1), in both serum and liver homogenates (Figures 6A,B). Consistent with these findings, reverse Transcription Quantitative Polymerase Chain Reaction (RT-qPCR) analysis showed a marked upregulation of proinflammatory genes (IFN-γ, TNF-α, IL-6, and inducible nitric oxide synthase [iNOS]) in the No-cell-18h post Con A group, which was significantly alleviated by the administration of LF-USC (Figure 6C).

Immunofluorescence staining further demonstrated that LF-USC treatment markedly reduced F4/80-positive macrophage infiltration and TNF-αexpression (Figure 6D). Quantitative assessment of TNF-α-positive hepatic regions confirmed a pronounced reduction in the LF-USC-72h post Con A group compared with the No-cell-18h post Con A group (Figure 6E). Moreover, Western blot analysis verified IL-6 downregulation following LF-USC therapy, while IL-10 expression patterns were consistent with serum measurements and RT-qPCR results (Figure 6F). Collectively, these findings indicate that LF-USCs mitigate hepatic injury in Con A-induced ALF by modulating inflammatory responses.

### 3.7 LF-USC transplantation gradually restores macrophage polarization toward an anti-inflammatory phenotype in injured livers

**FIGURE 7:**
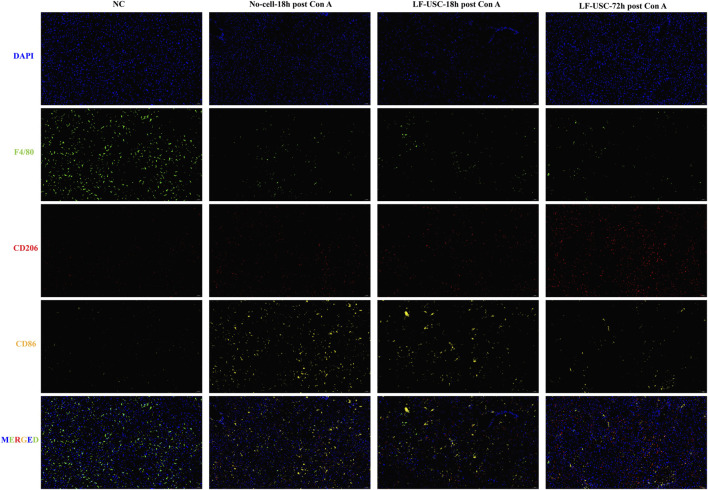
Immunofluorescence analysis of macrophage markers. Three mice were randomly selected from each group (n = 6). Representative staining of F4/80, CD206, and CD86 in liver tissues. NC livers showed abundant F4/80^+^ cells with low CD206 and minimal CD86. The No-cell-18 h post Con A group exhibited reduced F4/80 and CD206 and increased CD86. The LF-USC-18 h post Con A group showed a similar pattern to the No-cell-18 h post Con A group, whereas the LF-USC-72 h post Con A group displayed increased CD206 and reduced CD86, approaching NC levels. Scale bar = 50 μm. The magnification is ×200.

To assess the immunomodulatory effects of LF-USCs within the injured hepatic microenvironment, we performed triple immunofluorescence staining for F4/80, CD206, and CD86 across four experimental groups (Figure 7). In normal liver tissue, abundant F4/80^+^ macrophages were detected alongside low CD206 and minimal CD86 signals, reflecting a homeostatic macrophage landscape. In contrast, the No-cell-18 h post Con A group exhibited a pronounced loss of F4/80^+^ cells together with elevated CD86 and mildly decreased CD206 expression, indicative of a strong pro-inflammatory M1 response during acute injury.

Transplantation of LF-USCs at the 18 h time point did not immediately alter this inflammatory profile, as F4/80 remained low, CD206 showed only minimal recovery, and CD86 persisted at high levels, suggesting that early-phase macrophage polarization was largely unaffected. However, by 72 h post-transplantation, LF-USC-treated livers displayed a marked shift toward an anti-inflammatory phenotype, characterized by substantial accumulation of CD206^+^ macrophages and a notable reduction in CD86 expression, approaching levels observed in healthy tissue despite the incomplete restoration of F4/80^+^ cells. These temporal changes suggest that LF-USCs gradually redirect macrophage polarization toward a reparative M2 state while dampening M1-driven inflammation during injury resolution.

Interestingly, CD206 did not fully colocalize with F4/80, indicating the presence of phenotypically distinct CD206^+^ populations or macrophages at different activation states. Given the complexity of triple labeling and the partial non-colocalization of markers, this analysis was presented qualitatively.

### 3.8 Transcriptomic profiling reveals a shift from inflammatory injury to repair-associated programs following LF-USC treatment

**FIGURE 8:**
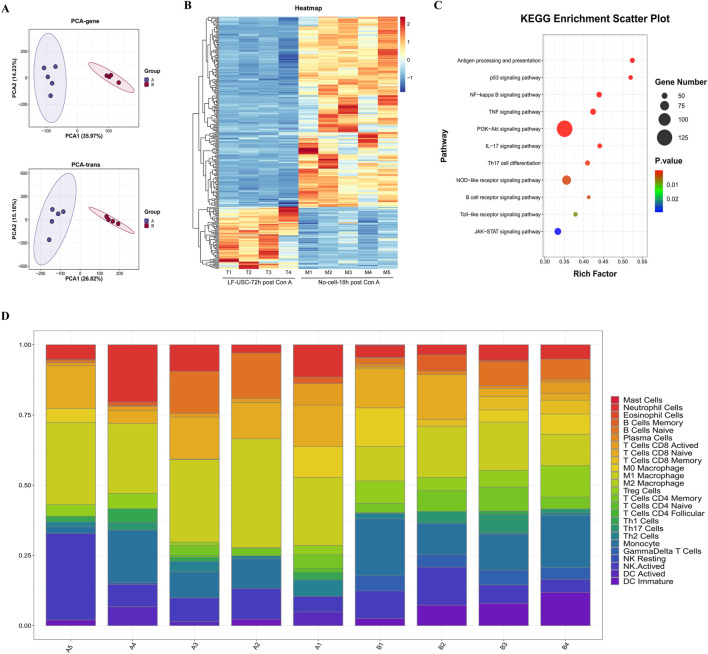
Transcriptomic profiling reveals that LF-USC treatment attenuates inflammation and modulates immune responses in Con A-induced acute liver injury. **(A)** Principal component analysis (PCA) of liver transcriptomes from the No-cell-18 h post Con A group (n = 5) and the LF-USC-72 h post Con A group (n = 4), showing distinct clustering. **(B)** Differentially expressed genes (DEGs) heatmap highlighting reduced inflammatory/apoptotic gene expression and increased cell cycle-related genes after LF-USC treatment. **(C)** KEGG enrichment of DEGs, with PI3K-Akt, TNF, NF-κB, antigen processing, and p53 pathways among the top hits. **(D)** Deconvolution analysis showing reduced M1-like macrophage infiltration in LF-USC-treated livers. A: the No-cell-18 h post Con A group; B: the LF-USC-72 h post Con A group.

To further elucidate the molecular basis underlying hepatocyte recovery following LF-USC therapy, transcriptomic profiling was performed on liver tissues from LF-USC-treated mice (72 h post Con A) and the No-cell group (18 h post Con A). Principal component analysis (PCA) demonstrated a clear separation along PC1 and PC2, indicating that LF-USC infusion substantially reshaped the hepatic transcriptional landscape (Figure 8A). Differential expression analysis further showed broad suppression of inflammation- and apoptosis-related genes (e.g., Ifnb1, Il1b, Tnf, Tlr4, Myd88, Nfkbia), accompanied by upregulation of key regulators of cell cycle progression and proliferation (e.g., Ccna2, Cdk1, Ccnb1/2, Mcm5), suggesting a shift from an injury-driven transcriptional state toward one favoring repair and regeneration (Figure 8B).

Pathway analyses reinforced this transition. KEGG enrichment highlighted PI3K-Akt, TNF, NF-κB, p53, and antigen processing and presentation as the most significantly affected pathways, with PI3K-Akt showing the strongest enrichment (Figure 8C). Consistently, GSEA revealed coordinated negative enrichment of multiple inflammation- and signaling-related pathways, including Hedgehog, IL-17, Jak-STAT, mTOR, NF-κB, PI3K-Akt, Toll-like receptor, TNF, and Wnt, while Hallmark analysis confirmed suppression of hypoxia response, IL-6/JAK/STAT3 signaling, interferon responses, mTORC1 signaling, TNFα/NF-κB signaling, MYC targets, oxidative phosphorylation, and the unfolded protein response (Supplementary Figures S1A,B). These transcriptional signatures aligned with the reduced levels of circulating IL-6 and TNF-α in LF-USC-treated mice.

Immune deconvolution analysis further revealed a marked reduction in pro-inflammatory M1 macrophages in the LF-USC group, although M1 macrophages remained the predominant myeloid subset in both groups (Figure 8D). Given the central function of NF-κB and MAPK signaling in driving M1 polarization, these findings suggest that LF-USCs mitigate hepatic inflammation, at least in part, by attenuating M1 macrophage activation.

Together, these transcriptomic data indicate that LF-USC transplantation shifts the hepatic response from an inflammation-dominated injury state toward a transcriptional program favoring tissue repair and hepatocyte regeneration.

### 3.9 LF-USCs transplantation showed no significant T cell infiltration, while macrophages persistently accumulated in the injured liver

**FIGURE 9:**
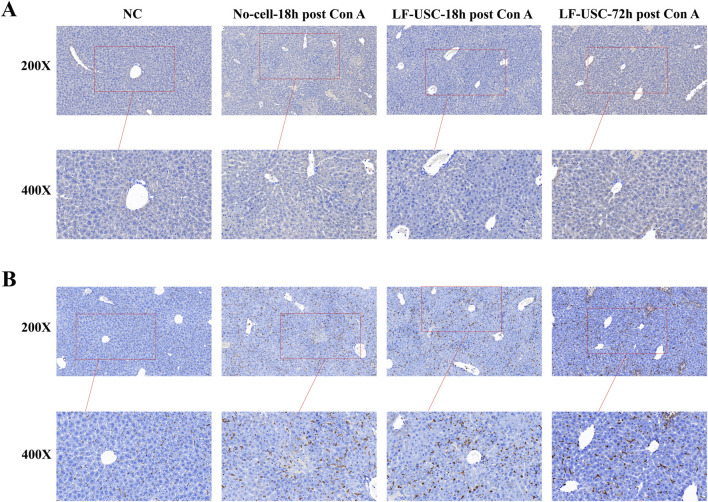
Immunohistochemical assessment of CD3^+^ T-cell infiltration and CD68^+^ macrophage accumulation in Con A-induced acute liver injury. **(A)** Representative immunohistochemical images of CD3 staining in liver tissues from the Normal, No-cell-18 h post Con A, LF-USC-18 h post Con A, and LF-USC-72 h post Con A groups. **(B)** Representative immunohistochemical images of CD68 staining. Scale bars = 50 μm.

Immunohistochemical staining for CD3 and CD68 was performed to evaluate immune cell infiltration in liver tissues across all four groups. The results revealed only a few scattered CD3^+^ T cells in the liver tissues of each group, with no significant CD3^+^ T cell aggregation or extensive infiltration observed (Figure 9A). This suggests that, within the early observation time window established in this study, xenotransplantation of LF-USCs did not induce significant rejection-related T cell infiltration changes at the local tissue level in the liver. In contrast, CD68^+^ macrophages were readily detected in all groups and exhibited markedly increased abundance in the No-cell-18 h post Con A, LF-USC-18 h post Con A, and LF-USC-72 h post Con A groups compared with normal controls (Figure 9B). This heightened CD68 signal reflects robust monocyte–macrophage recruitment during acute hepatic injury, consistent with the inflammatory response elicited by Con A. Notably, macrophage accumulation remained evident in LF-USC-treated mice, corroborating the dynamic polarization patterns revealed by immunofluorescence analysis.

## 4 Discussion

In this study, we successfully isolated and expanded LF-USCs from the urine of ACLF patients and systematically characterized their biological properties. *In vitro* analyses revealed that LF-USCs displayed a typical mesenchymal stem cell phenotype, characterized by stable adherent growth, positive expression of MSC-specific surface markers (CD73, CD90, and CD105), absence of hematopoietic stem cell markers (CD34, CD45, and CD11b), and robust proliferative capacity as well as multipotent differentiation potential (Yu et al., 2024). These findings indicate that, under the experimental conditions examined, LF-USCs retain key stem cell-like characteristics despite originating from donors with profound hepatic dysfunction, supporting their feasibility as a patient-derived cell source for regenerative applications.

Notably, LF-USCs maintained proliferative capacity comparable to healthy donor-derived USCs during early passages (up to P7), which is particularly relevant for translational exploration. Early-passage expansion enables the generation of sufficient cell numbers while including properties commonly associated with paracrine activity and immunomodulation, which may deteriorate with extended passaging. These results suggest that LF-USCs obtained from ACLF patients are amenable to short-term *in vitro* expansion for preclinical evaluation, despite the underlying inflammatory and metabolic stress associated with liver failure.

To comprehensively evaluate the *in vivo* reparative capacity of LF-USCs, we employed a Con A-induced acute liver injury mouse model for cell transplantation experiments (Yang et al., 2025). Con A is a plant-derived lectin that activates T cells and triggers a robust immune response, leading to pathological alterations resembling human immune-mediated acute liver injury, including extensive hepatocellular necrosis, cytokine storm, and severe hepatic dysfunction (Greco et al., 2016). Owing to these features, the Con A-induced acute liver injury model has been widely applied to elucidate the mechanisms of immune-mediated liver injury and to assess the therapeutic efficacy of stem cell-based interventions (Yang et al., 2025; Yang et al., 2022; Liu Q. et al., 2021). Further analyses demonstrated that LF-USC therapy was associated with attenuation of liver injury, as reflected by reductions in serum transaminase and bilirubin levels, decreased hepatocellular necrosis and apoptosis, restoration of histological architecture, replenishment of glycogen storage, and attenuation of fibrosis, consistent with the therapeutic effects reported in previous stem cell-based studies (Lu et al., 2025; Zhang et al., 2025). These protective effects were evident during the early stage of acute injury and became more pronounced at 72 h, indicating a time-dependent contribution of LF-USCs to liver repair.

In addition, the protective effects of LF-USCs in the acute liver injury mouse model were primarily mediated through modulation of inflammatory responses. Con A treatment markedly elevated several proinflammatory cytokines (e.g., IFN-γ, IL-6, TNF-α, IL-1β, MCP-1), reflecting severe immune-mediated hepatic injury (Feng et al., 2018; Xiao et al., 2025). Following LF-USC transplantation, inflammatory activity was partially suppressed at 18 h, and cytokine levels were substantially reduced by 72 h, demonstrating a time-dependent anti-inflammatory effect. Notably, IL-10 was significantly upregulated at 18 h, suggesting that LF-USCs counteract acute inflammation by enhancing anti-inflammatory cytokine secretion; as inflammation subsided, IL-10 levels gradually returned toward baseline (Zhang et al., 2021; Luan et al., 2021). TGF-β levels also rose during the early treatment phase, potentially reflecting roles in tissue repair and initiation of fibrosis, but subsequently declined during recovery, indicating that LF-USCs promote repair while preventing excessive fibrogenesis (Frangogiannis, 2020). Immunofluorescence and Western blot analyses further confirmed that LF-USCs attenuate macrophage activation and reduce proinflammatory cytokine release, thereby mitigating liver injury. Taken together, these findings suggest that LF-USC transplantation is associated with dynamic, biphasic modulation of inflammatory responses in Con A-induced acute liver injury: suppressing excessive inflammation and promoting repair in the early phase, while maintaining immune homeostasis and limiting fibrosis during recovery.

Macrophage polarization emerged as a prominent immunological feature associated with LF-USC treatment. Immunofluorescence analyses showed a predominance of CD86^+^ M1-like macrophages during the early phase of liver injury, followed by an increased presence of CD206^+^ M2-like macrophages at 72 h, while overall macrophage abundance (F4/80^+^ cells) was partially restored. These findings suggest that LF-USC treatment is associated with alterations in macrophage functional states rather than a marked inhibition of macrophage recruitment. Transcriptomic profiling further supported this interpretation by revealing a coordinated downregulation of inflammation and apoptosis-related genes and signaling pathways, including TNF, NF-κB, IL-6/JAK-STAT3, Toll-like receptor, and PI3K-Akt pathways, together with relative enrichment of gene programs linked to cell cycle progression and tissue repair. Although these transcriptomic changes are descriptive in nature, their consistency with histological and immunophenotypic findings supports a model in which LF-USC treatment is associated with a shift in the hepatic immune microenvironment toward a more reparative state. Collectively, these observations indicate that LF-USCs are associated with remodeling of the hepatic immune landscape in immune-mediated liver injury. This effect is consistent with the reported paracrine and immunomodulatory properties of mesenchymal stem-like cells, while further mechanistic studies will be required to define the specific molecular mediators involved (Yang et al., 2023; Alvites et al., 2022).

In addition, the absence of CD3^+^ T-cell infiltration across all groups indicates that xenotransplantation of human LF-USCs does not provoke measurable early adaptive immune responses within the first 72 h. This observation is consistent with the delayed kinetics of T-cell-mediated xenogeneic rejection and suggests that the observed protective effects occur before overt adaptive immune activation (Li and Lan, 2023). In contrast, the consistently elevated CD68 expression in Con A-treated groups reflects rapid recruitment of monocyte-derived macrophages during acute hepatic inflammation, a defining feature of Con A-induced injury (Hao et al., 2022). The persistence of macrophages alongside a phenotypic shift from M1-to M2-like states further supports the interpretation that LF-USCs mitigate liver injury primarily by modulating inflammatory signaling and macrophage function rather than by inducing early immune tolerance or blocking immune cell infiltration.

Several limitations of this study should be acknowledged. First, although the Con A-induced mouse model recapitulates key features of immune-mediated acute liver injury, it does not fully reflect the complexity and heterogeneity of human acute-on-chronic liver failure. Second, LF-USCs were derived from a small number of ACLF patients (n = 3), all of whom were male and had HBV-related disease, which precluded systematic evaluation of donor-dependent variability related to etiology, sex, age, or disease severity. Accordingly, the present findings should be regarded as preliminary. Third, this study did not include a direct *in vivo* comparison between LF-USCs and urine-derived stem cells from healthy donors. While such comparisons would provide additional insight into donor disease–related effects and autologous *versus* allogeneic immunological influences, the current experimental design focused on determining whether USCs obtained from a profoundly inflammatory disease background retain immunomodulatory and hepatoprotective capacity under conditions of immune-mediated acute liver injury. Future studies incorporating healthy donor–derived USCs will be important to further clarify these issues. Finally, human LF-USCs were transplanted into immunocompetent C57BL/6 mice, and although no overt CD3^+^ T-cell infiltration was observed within the early observation window, this does not exclude the possibility of delayed xenogeneic immune responses. Longer-term studies, extended immune monitoring, and the use of humanized or immunomodulatory models will be required to more fully assess immune compatibility, cell fate, and the durability of LF-USC-based therapy.

In conclusion, this study provides preclinical evidence supporting the potential of USCs isolated from ACLF patients as a therapeutic approach for acute liver injury. Transplantation of high-concentration RGD-modified hydrogel-encapsulated LF-USCs significantly improved animal survival and attenuated liver damage in a murine ALF model, while also suppressing systemic inflammation—a critical driver of disease progression. Furthermore, LF-USCs can be collected non-invasively and repeatedly from patients, thereby reducing ethical concerns and minimizing the risk of immune rejection. These advantages position LF-USCs as a clinically attractive, personalized cell source for early intervention in acute liver injury and for preventing progression to ACLF. To translate these findings into clinical practice, future studies should focus on validating the safety, scalability, and therapeutic efficacy of LF-USCs in patients with liver failure.

## Data Availability

The RNA-seq data generated in this study have been deposited in the NCBI Sequence Read Archive (SRA) under BioProject accession number PRJNA1447582.
