# Supplementary material for: Functional characterization of urine-derived stem cells from acute-on-chronic liver failure patients in an immune-mediated acute liver injury model
Source: Front Bioeng Biotechnol. 2026 Apr 17;14:1759241. doi: 10.3389/fbioe.2026.1759241 (PMC13133080; doi:10.3389/fbioe.2026.1759241)
Supplement: Supplementary file 1 [file Supplementaryfile1.docx]

Supplementary Material

# Supplementary Tables

**Supplementary Table 1.** Primary baseline clinical characteristics of ACLF patients.

| Number | Number-1 | Number-2 | Number-3 | Normal arranges |  |
| --- | --- | --- | --- | --- | --- |
| Age | 34 | 49 | 23 |  |  |
| Gender | Male | Male | Male |  |  |
| EASL-CLIF  ACLF grade | Grade 2 | Grade 3 | Grade 2 |  |  |
| Primary cause | HBV | HBV | HBV |  |  |
| HGB(g/L) | 160 | 118 | 146 | 130–175 |  |
| WBC(10^9/L) | 10.31 | 14.9 | 15.7 | 3.5–9.5 |  |
| PLT(10^9/L) | 94 | 35 | 40 | 125–350 |  |
| NLR | 4.3663 | 6.2308 | 8.5973 | 1–3 |  |
| INR | 2.65 | 2.87 | 3.65 | 0.8–1.2 |  |
| ALT(U/L) | 279 | 46 | 89 | 9–50 |  |
| AST(U/L) | 342 | 59 | 41 | 15–40 |  |
| TBIL(μmol/L) | 514.5 | 278.1 | 328.6 | 5.1–19 |  |
| ALB(g/L) | 32.1 | 29.1 | 29.4 | 40–55 |  |
| ALP(U/L) | 184 | 106 | 189 | 45–125 |  |
| γ-GT(U/L) | 76 | 23 | 27 | 10–60 |  |
| UREA(mmol/L) | 6.13 | 25.59 | 5.8 | 2.6–7.5 |  |
| CREA(μmol/L) | 55 | 189 | 51 | 62–115 |  |
| eGFR | 129.2 | 35.1 | 144 | ≥90 |  |
| Urinary leukocytes  (per high-power field) | 0 | 0 | - | 0–5 |  |
| RBC in urine  (per high-power field) | 0 | 3 | - | 0–3 |  |
| Urine Specific Gravity | 1.015 | 1.02 | - | 1.005–1.030 |  |
| Na(mmol/L) | 133.4 | 128 | 132.3 | 135–145 |  |
| CHOL(μmol/L) | 2.29 | 1.43 | 2.12 | < 5.2 mmol/L |  |
| TG(μmol/L) | 1.99 | 0.94 | 0.85 | < 1.7 mmol/L |  |
| CRP(μg/ml) | 2.6 | 61.1 | - | < 10 |  |
| **Abbreviations:** ACLF, Acute-on-chronic liver failure; ACLF grading was performed according to the EASL-CLIF Consortium criteria; HGB, Hemoglobin; WBC, White Blood Cell; PLT, Platelet; NLR, Neutrophil-to-Lymphocyte Ratio; INR, International Normalized Ratio; ALT, Alanine Aminotransferase; AST, Aspartate Aminotransferase; TBIL, Total Bilirubin; ALB, Albumin; ALP, Alkaline Phosphatase; γ-GT, Gamma-Glutamyl Transferase; CREA, Creatinine; eGFR, Estimated Glomerular Filtration Rate; RBC, Red Blood Cell; CHOL, Cholesterol; TG, Triglyceride; CRP, C-Reactive Protein. | | | | |  |
| **Supplementary Table 2.** Antibodies used for flow cytometric characterization of USCs.   \| Marker \| Source \| Catalog No. \| Working Condition \| Purpose \| \| --- \| --- \| --- \| --- \| --- \| \| CD90 \| Bioss \| bsm-30105M \| 1:50 \| MSC positive marker \| \| CD73 \| OriCell^®^ Human MSC Surface Marker Analysis Kit^[1-3]^ \| HUXMX-09011 \| 2 µL / 3×10⁵ cells \| \| CD105 \| \| CD34 \| Hematopoietic exclusion \| \| CD45 \| \| CD11b \| \| Mouse IgG1 κ \| Negative control \| \| Rat IgG2b κ \| \| Goat Anti-Mouse IgG-FITC \| 2 µL \| Detection \| \| Goat Anti-Mouse IgG-PE \| \| Goat Anti-Rat IgG-FITC \| \| Goat Anti-Rat IgG-PE \| \| **Note:** The OriCell^®^ Human MSC Surface Marker Analysis Kit is a commercially validated assay. The staining procedure in this study was performed strictly according to the manufacturer’s instructions. Selected technical references listed in the product manual are provided in the reference list for background information on marker validation and application of this assay. \| \| \| \| \| | | | | |  |

**Supplementary Table 3.** Specific Information on the ELISA Kits.

| ELISA kit | Concentrations | Manufacturer | Catalog No. |
| --- | --- | --- | --- |
| Mouse IL-10 Uncoated ELISA Kit | 1:250 | Thermo Fisher Scientific | 88-7105-88 |
| TNF-α Mouse Uncoated ELISA Kit | 1:250 | Thermo Fisher Scientific | 88-7324-88 |
| Mouse IL-6 ELISA Kit | 1:250 | Thermo Fisher Scientific | 88-7064-88 |
| Mouse IFN-γ ELISA Kit | 1:100 | Servicebio | GEM0006 |
| Mouse IL-1β Uncoated ELISA Kit | 1:250 | Thermo Fisher Scientific | 88-7013A-88 |
| Mouse CCL2/MCP-1 ELISA Kit | 1:250 | Thermo Fisher Scientific | 88-7391-88 |
| Mouse/Human TGF-β ELISA Kit | 1:250 | Thermo Fisher Scientific | 88-8350-88 |
| **Abbreviations:** IL-10, Interleukin-10; TNF-α, Tumor Necrosis Factor-α; IL-6, Interleukin-6; IFN-γ, Interferon-gamma; IL-1β, Interleukin-1 beta; CCL2/MCP-1, C-C motif chemokine ligand 2 / Monocyte Chemoattractant Protein-1; TGF-β, Transforming Growth Factor-beta. | | | |

**Supplementary Table 4.** Antibodies used for immunofluorescence and immunohistochemistry.

| Marker | Dilution | Catalog Number | Manufacturer |
| --- | --- | --- | --- |
| F4/80 | 1:5000 | GB113373 | Servicebio |
| TNF-α | 1:5000 | GB115702 |  |
| CD206 | 1:4000 | GB113497 |  |
| CD86 | 1:5000 | GB150054 |  |
| HRP-conjugated goat anti-rabbit IgG | 1:500 | GB23303 |  |
| CD3 | 1:1000 | GB150004 |  |
| CD68 | 1:3000 | GB153109 |  |
| S-vision Polyclonal Antibody (Goat Anti-Rabbit) | Ready-to-use | G1302 |  |

**Supplementary Table 5.** Primer sequences (5′-3′) used in real time RT-qPCR.

| Gene | Primer sequences (5′→3′) | Manufacturer |
| --- | --- | --- |
| m GAPDH | AACTTTGGCATTGTGGAAGG (F) | Sangon Biotech |
|  | ACACATTGGGGGTAGGAACA (R) |  |
| IFN-γ | CTGGAGGAACTGGCAAAAGGATGG (F) |  |
|  | GACGCTTATGTTGTTGCTGATGGC (R) |  |
| TNF-α | GCCTCTTCTCATTCCTGCTTGTGG (F) |  |
|  | GTGGTTTGTGAGTGTGAGGGTCTG (R) |  |
| IL-6 | CTTCTTGGGACTGATGCTGGTGAC (F) |  |
|  | AGGTCTGTTGGGAGTGGTATCCTC (R) |  |
| iNOS | ACTCAGCCAAGCCCTCACCTAC (F) |  |
|  | TCCAATCTCTGCCTATCCGTCTCG (R) |  |
| IL-10 | TCCCTGGGTGAGAAGCTGAAGAC (F) |  |
|  | CACCTGTCCACTGCCTTGC (R) |  |
| TGF-β | ATCTGTGAGAAGCCGCATGAAGTC (F) |  |
|  | AGAGTGAAGCCGTGGTTAGGTGAG (R) |  |

**Supplementary Table 6.** List of antibodies used for Western blotting in this study.

| A list of primary antibodies | | | | | | | | | |
| --- | --- | --- | --- | --- | --- | --- | --- | --- | --- |
| Target Protein | Manufacturer | Catalog No. | | Molecular Weight (kDa) | | Host Species | | Dilution |  |
| ACTIN | Servicebio | GB12001 | | 42 | | Mouse | | 1: 2000 |  |
| IL-6 | Servicebio | GB11117-100 | | 21-24 | | Rabbit | | 1: 500 |  |
| IL-10 | Servicebio | GB11108-100 | | 15-25 | | Rabbit | | 1: 500 |  |
| A list of secondary antibodies | | | | | | | | | |
| Name | | | Manufacturer | | Catalog No. | | Dilution | | |
| HRP-conjugated goat anti-mouse | | | Servicebio | | GB23301 | | 1：5000 | | |
| HRP-Goat Anti-Rabbit | | | Servicebio | | GB23303 | | 1：3000 | | |
| HRP-Goat Anti-Rabbit | | | Servicebio | | GB23303 | | 1：3000 | | |
| **Note:** In the order of the tables above, each primary antibody corresponds to its respective secondary antibody. | | | | | | | | | |

# Supplementary Methods

**Supplementary Method 1. Subculture and Expansion of Urine-Derived Stem Cells (USCs)**

1. **Subculture Initiation:** Approximately two weeks after primary culture, when large or multiple colonies became visible in the well plates, the culture medium was carefully aspirated using a pipette, ensuring maximal removal while avoiding disturbance of the adherent cells.
2. **Cell Washing:** The cell layer was gently rinsed 2-3 times with 1× phosphate-buffered saline (PBS) to remove residual medium. After complete aspiration of PBS, pre-warmed (37°C) 0.25% Trypsin-EDTA was added to each well at an appropriate volume, and the plate was incubated at 37°C in 5% CO_2_ for 3-5 minutes. Cell detachment was monitored every 30 seconds under an inverted microscope.
3. **Neutralization:** Once the cells became rounded and began to detach, enzymatic digestion was immediately neutralized by adding serum-containing USC culture medium at a volume three times that of the Trypsin-EDTA. The plate was gently swirled to ensure uniform mixing.
4. **Cell Collection:** Cells were gently pipetted to achieve complete detachment and generate a single-cell suspension, which was transferred to a 15 mL centrifuge tube. Suspensions from multiple wells were combined when necessary.
5. **Centrifugation:** The collected cells were centrifuged at 1000 rpm for 5 minutes at 4°C. The supernatant was carefully discarded without disturbing the cell pellet.
6. **Resuspension:** The cell pellet was resuspended in 1 mL of pre-warmed (37°C) USC culture medium by gentle pipetting to ensure even dispersion.
7. **Plating and Subsequent Culture:** The resuspended cells were seeded into a T25 culture flask containing 9 mL of pre-warmed USC culture medium and designated as passage 1 (P1). Cells were maintained under standard culture conditions (37°C, 5% CO₂) and the medium was replaced every two days. Upon reaching 80%-90% confluence, cells were passaged using the procedure described above. For routine expansion, cells were reseeded at a density of 1.0 × 10^4^ cells/cm^2^ until required for downstream experiments.

# Supplementary Figures

**Supplementary Figure 1.** Pathway enrichment and immune infiltration changes following LF-USC treatment. (A) GSEA enrichment plots showing broad suppression of inflammatory and stress-related pathways—including IL-17, JAK-STAT, TNF, NF-κB, PI3K-Akt, mTOR, Toll-like receptor, p53 and Wnt signaling—in LF-USC–treated livers (all ES < 0). (B) Hallmark gene sets further demonstrate coordinated downregulation of hypoxia, IL6–JAK–STAT3, interferon responses, mTORC1, MYC targets, oxidative phosphorylation, protein secretion and TNFα–NF-κB signaling after LF-USC therapy. A: the No-cell-18 h post Con A group; B: the LF-USC-72 h post Con A group.

**
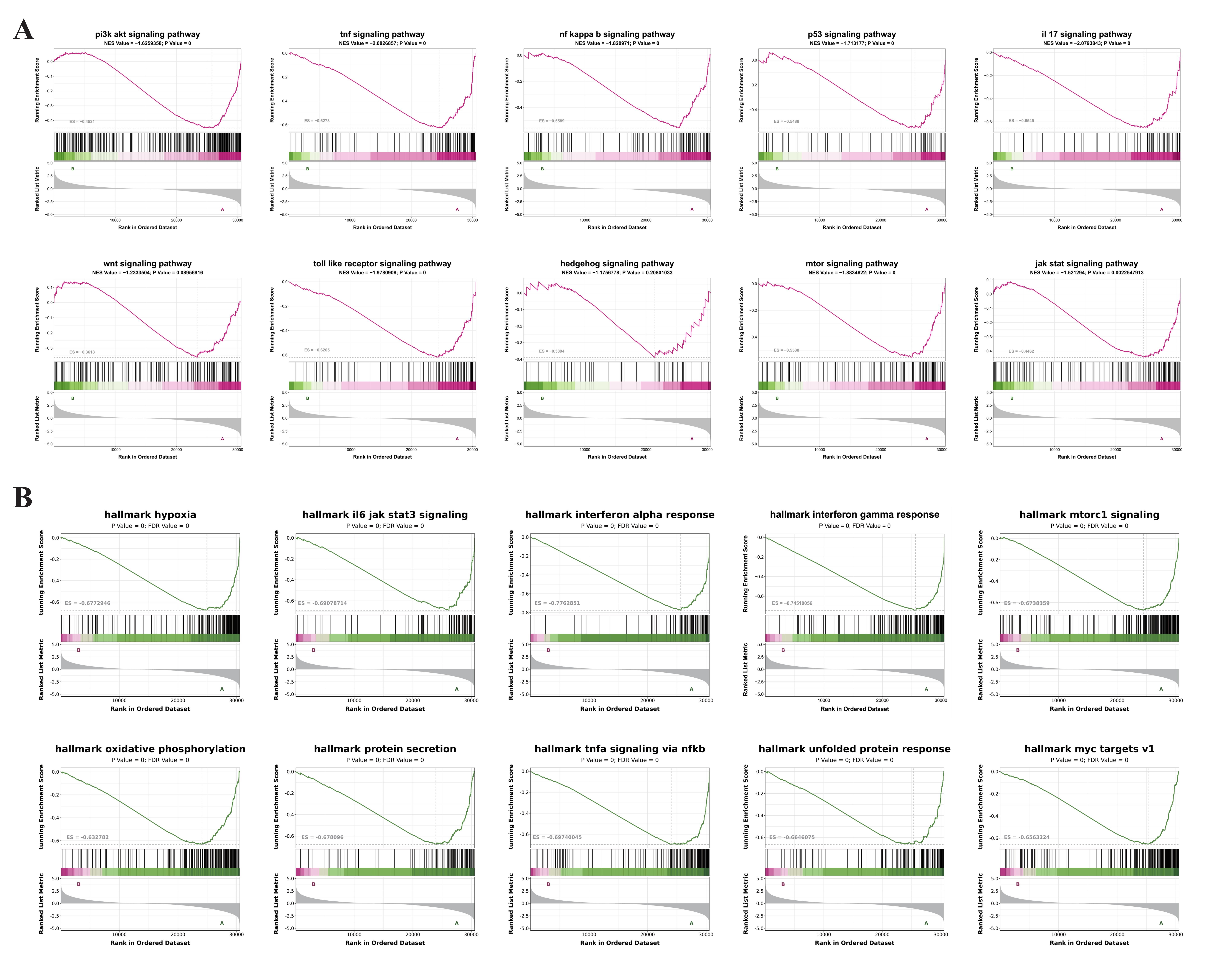
**

**Reference**

[1] PROCKOP DJ. Marrow Stromal Cells as Stem Cells for Nonhematopoietic Tissues[J]. Science, 1997, 276(5309): 71-4. DOI: 10.1126/science.276.5309.71.

[2] PITTENGER MF, MACKAY AM, BECK SC, et al. Multilineage Potential of Adult Human Mesenchymal Stem Cells[J]. Science, 1999, 284(5411): 143-7. DOI: 10.1126/science.284.5411.143.

[3] HUMPHREYS BD, BONVENTRE JV. Mesenchymal Stem Cells in Acute Kidney Injury[J]. Annu Rev Med, 2008, 59: 311-25. DOI: 10.1146/annurev.med.59.061506.154239.
